# Characterization and history of arterial hypertension leading to inpatient treatment

**DOI:** 10.1186/s13104-016-2285-y

**Published:** 2016-10-24

**Authors:** Carsten P. Bramlage, Mina Nasiri-Sarvi, Joan Minguet, Peter Bramlage, Gerhard Anton Müller

**Affiliations:** 1 Institute for Pharmacology and Preventive Medicine (IPPMED), Bahnhofstr. 20, 49661 Cloppenburg, Germany; 2 Department of Nephrology and Rheumatology, Georg-August-University of Goettingen, Robert-Koch Street 40, 37075 Goettingen, Germany

**Keywords:** Arterial hypertension, Inpatient, Patient characteristics, Treatment

## Abstract

**Background and aims:**

Arterial hypertension is a major cause of death worldwide. For the most part, treatment for hypertension can be performed on an outpatient basis. However, some patients also require inpatient treatment, and the contributing factors for this remain unknown. Therefore, the primary objective of the present study was to determine which patient characteristics are associated with inpatient treatment for arterial hypertension.

**Methods:**

Here, we conducted a mono-centric study of 103 hypertensive subjects, who were treated as inpatients in the Department of Nephrology and rheumatology of the university medical faculty of Göttingen. Therapies were not altered, and data collection was performed retrospectively. In addition to epidemiological information, the following data were recorded: patient symptoms, blood pressure (BP), anti-hypertensive therapy, and concomitant diseases (e.g., renal and cardiovascular conditions).

**Results:**

Approximately half (53 %) of all subjects treated on an inpatient basis displayed elevated BP (>140/90 mmHg), while the remaining 47 % of patients showed normotensive readings (<140/90 mmHg) following admission. Moreover, 34 % of patients could be classified as therapy refractory. The main reasons for hospital admission were hypertension-related symptoms, including shortness of breath, dizziness, and headache (69 %). These patients were multi-morbid, with approximately 60 % displaying a secondary form of hypertension. Indeed, over half of the subjects showed renoparenchymatous forms of hypertension, and a large percentage of patients received hypertension-inducing drugs (32 %). Moreover, a high proportion of inpatients were treated with reserve antihypertensives, with the most commonly used drug being Moxonidin.

**Conclusion:**

The majority of hypertensive patients were hospitalized due to their clinical symptoms and not as a result of BP values alone. The high proportion of patients with secondary forms of hypertension or treated with BP-boosting medications was striking.

## Background

Arterial hypertension is a chronic medical condition in which blood pressure (BP) is elevated. It significantly increases the risk of heart disease, stroke, and renal insufficiency and is therefore a major cause of death globally [1, 2]. In spite of recent therapeutic advances, blood pressure control remains suboptimal [3]. This is especially true for cases involving serious comorbid conditions (e.g., kidney disease) [4]. Therefore, achieving effective therapeutic intervention is fundamental, and ongoing investigation into the treatment of hypertensive patients is essential for ensuring successful clinical management.

Although hypertension is not usually accompanied by symptoms, patients with severely elevated BP can experience effects (e.g., headaches, lightheadedness, vertigo, and/or fainting episodes) [5]. However, it remains controversial whether such manifestations are directly linked to high BP or not [6–8]. Notably, symptoms can also be associated with secondary hypertension, which results from known underlying causes (e.g., kidney disease, endocrine abnormalities, or medications) [9, 10]. Thus, irrespective of the etiologic basis, hypertension-associated symptoms can lead patients to pursue medical treatment.

For the most part, clinical management of hypertension is performed on an outpatient basis through lifestyle modification and/or various antihypertensive drugs. However, hypertension can be treatment refractory [11]. In this regard, there are various classes of therapies that can be combined to optimize treatment [12], including reserve antihypertensive drugs (e.g., Moxonidin, Ebrantil). Nevertheless, treating patients with resistant hypertension can be complex, and its impact remains to be fully elucidated [13].

Recent studies have suggested that during hospitalization uncontrolled BP is highly prevalent in hypertensive patients [14]. Thus, some individuals ultimately require inpatient treatment for elevated BP. However, the factors contributing to this and the benefit of such therapy are uncertain. Therefore, the objective of this study was to determine which patient characteristics are associated with the requirement for inpatient treatment of hypertension.

## Methods

### Study design and patients

The present study was a mono-centric investigation involving 103 patients with arterial hypertension, who were treated as inpatients in the Department of Nephrology at the University Medical Faculty of Goettingen (Germany). Therapies were not altered during the study, which included all patients with arterial hypertension admitted to the Department of Nephrology in December 2005. Moreover, this study was conducted in accordance with the Declaration of Helsinki. According to the guidelines of the ethics committee of the University Goettingen, this study did not require ethics approval since (a) the data collection were performed retrospectively, (b) therapies were not altered and (c) individual patient data are not transferred outside the university. All patients in the study provided informed consent for participation, in line with the contract concerning medical treatment of the University Goettingen.

### Data collection

Data were collected retrospectively by reviewing documentation made in patients’ charts. In addition to epidemiological data (i.e., age, gender, height, weight, body mass index [BMI]), the following information was recorded: patient symptoms (i.e., headaches, nosebleeds, dizziness, angina pectoris, nausea, blurred vision, night sweats, heart palpitations, shortness of breath); BP (following admission to the ward and upon discharge); anti-hypertensive medications; and concomitant diseases (i.e., hyperthyroidism, pheochromocytoma, Cushing’s syndrome, Conn’s syndrome, renoparenchymatous hypertension, sleep apnea syndrome, renal artery stenosis). Also, we collected information with regard to renal function (i.e., creatinine, proteinuria [yes/no], level of CKD, dialysis [yes/no]) and cardiovascular disease/risk factors (i.e., myocardial infarction, coronary heart disease (CHD), diabetes mellitus, hyperlipidemia, smoking). ECGs were performed at hospital admission. The dataset of the current study is available from the corresponding author on reasonable request.

### Statistical analysis

Continuous variables were summarized using descriptive statistics and are shown as means with standard deviations, whereas categorical data are presented as the percentage (%) of subjects within each category.

## Results

Here, we have conducted a mono-centric study of 103 patients, who were treated as inpatients for arterial hypertension. Therapies were not altered, and data collection was performed retrospectively. The primary objective of this investigation was to determine which patient characteristics are associated with the requirement for inpatient treatment for arterial hypertension.

### Patient characteristics

The epidemiological characteristics of our study population are presented in Table 1. These inpatients displayed a mean age of approximately 64 years and were approximately equally distributed with regard to sex. In addition, the average BMI for these subjects was 26.4, which indicated an overall normal weight. Moreover, on average, they had experienced arterial hypertension for 12 years based on time of diagnosis.

**Table 1:** Patient characteristics

	All patients (n = 103)
Age (years)	63.8 ± 14.8
Sex (% female)	49
BMI (kg/m^2^)	26.4 ± 4.8
Time of Diagnosis (years)	12.1 ± 8.4

*BMI* body mass index

### BP values on the day of admission and discharge

**Fig. 1:**
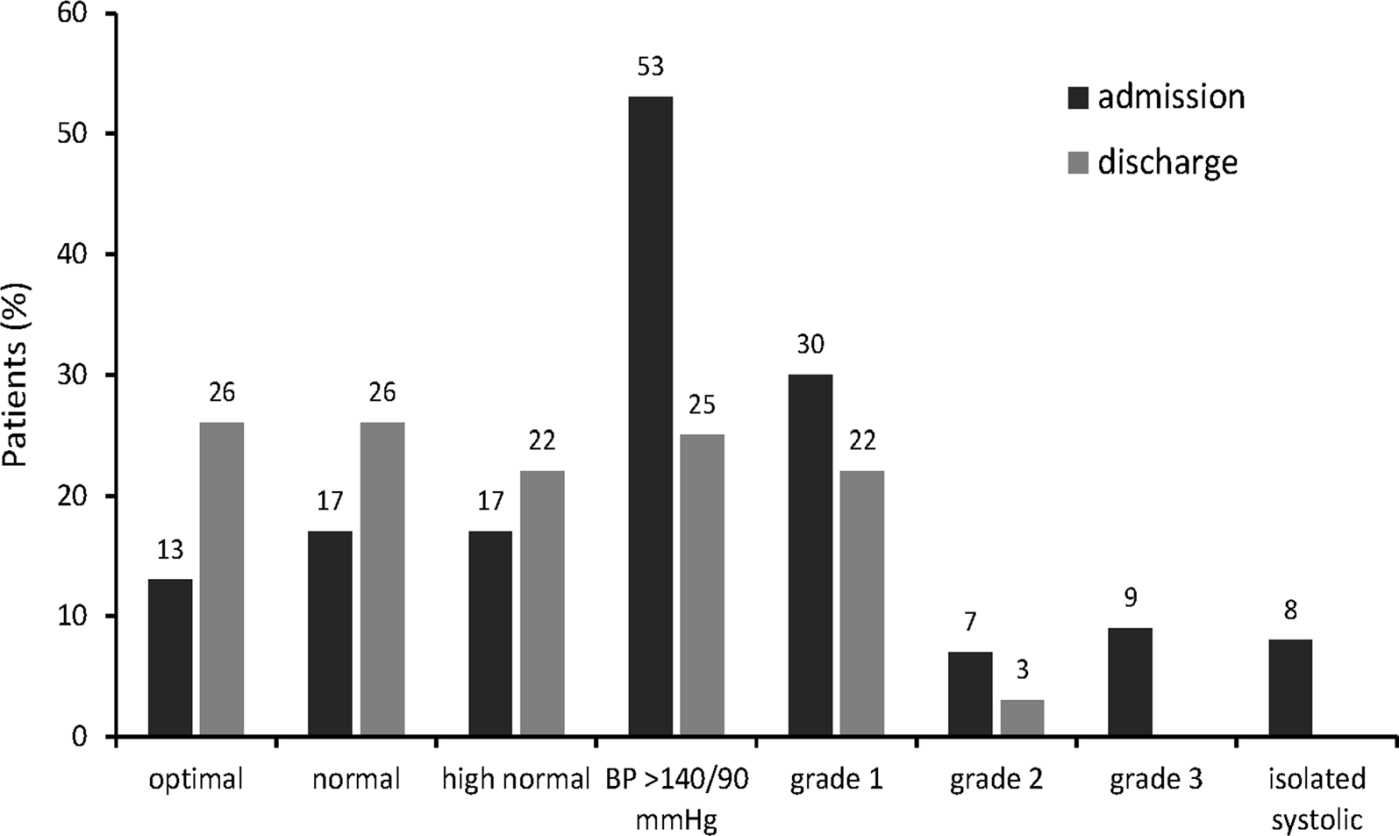
Distribution of patients base pressure (BP) category on the day of admission and at discharge

We assessed the BP of our inpatient population after admission to the hospital ward and found surprisingly low values. In fact, almost half of all patients (47 %) exhibited BP values below 140/90 mmHg, with only 9 % of the subjects displaying grade 3 hypertension (Fig. 1). In order to determine whether inpatient care could lead to improvements in these BP measurements, we also performed BP readings on the day of discharge. Indeed, 74 % of all patients had BP levels below the limit of 140/90 mmHg at the time of discharge, and there were no longer individuals presenting grade 3 hypertension (Fig. 1). Thus, subjects treated in the hospital for arterial hypertension collectively showed lower BP values than expected, and BP reduction could be effectively achieved through inpatient care.

### Patient characterization based on reason for admission

**Fig. 2:**
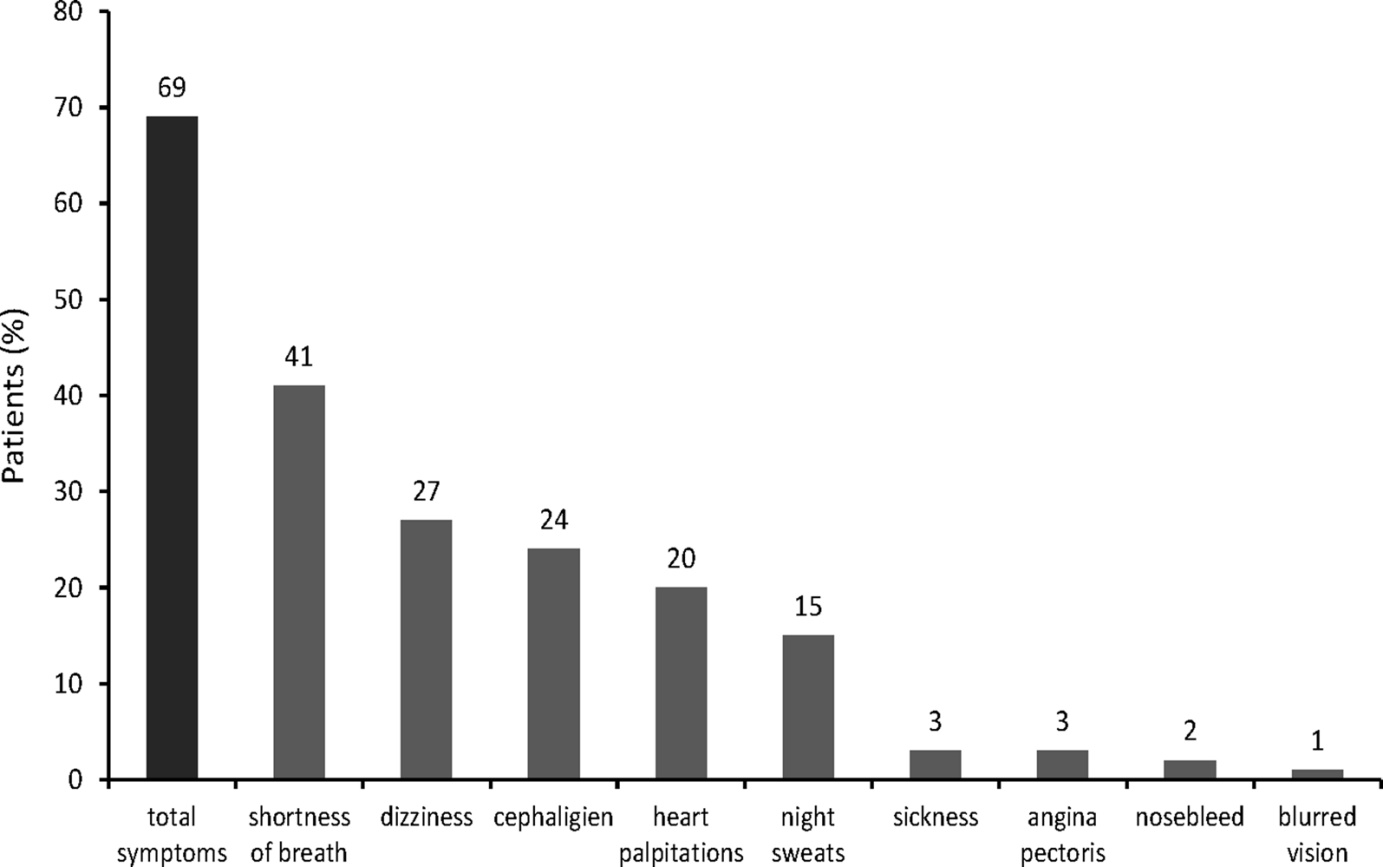
Distribution of hypertension-related symptoms upon hospital admission

Since high BP was the basis for hospitalization for only a few cases in our cohort, we next analyzed the overall reasons for hospital admission in our patients. In this regard, we found that symptoms caused either directly or indirectly by hypertension were the reason for admission in 68.9 % of the patients. Notably, the most common symptom that was reported was shortness of breath, which was followed by dizziness, headaches, and heart palpitations (Fig. 2).

### Proportion of patients with secondary hypertension

**Fig. 3:**
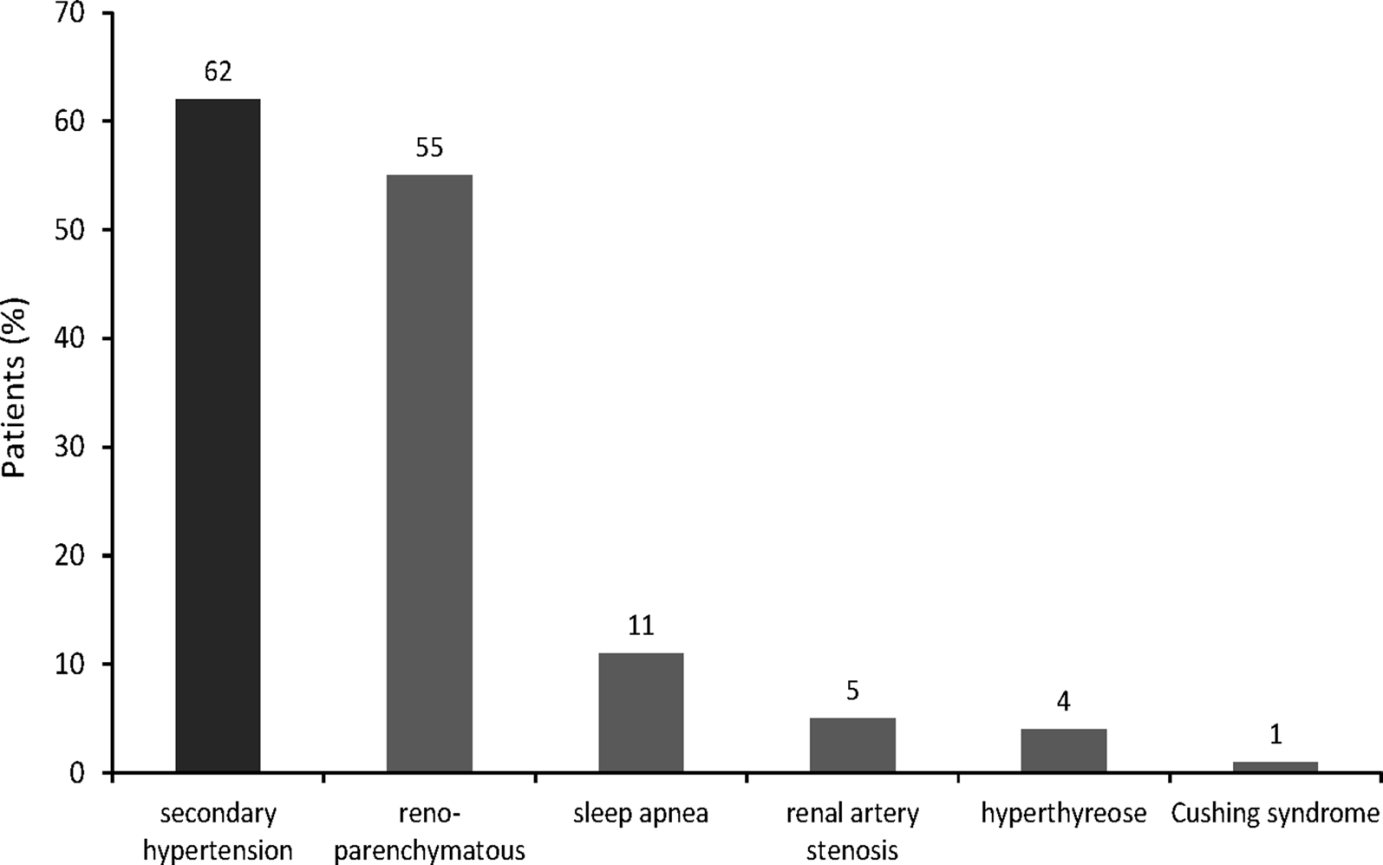
Distribution of patients with secondary hypertension

Among the patients hospitalized due to hypertension in the Department of Nephrology and Rheumatology, over 60 % were found to suffer from secondary forms of hypertension. In particular, more than half of the patients displayed renoparenchymatous hypertension. The overall frequency and causes of secondary hypertension in our patient population are presented in Fig. 3. Notably, secondary forms of hypertension that are not listed here (e.g., pheochromocytoma) were not observed in the present study.

### BP increasing medication

**Fig. 4:**
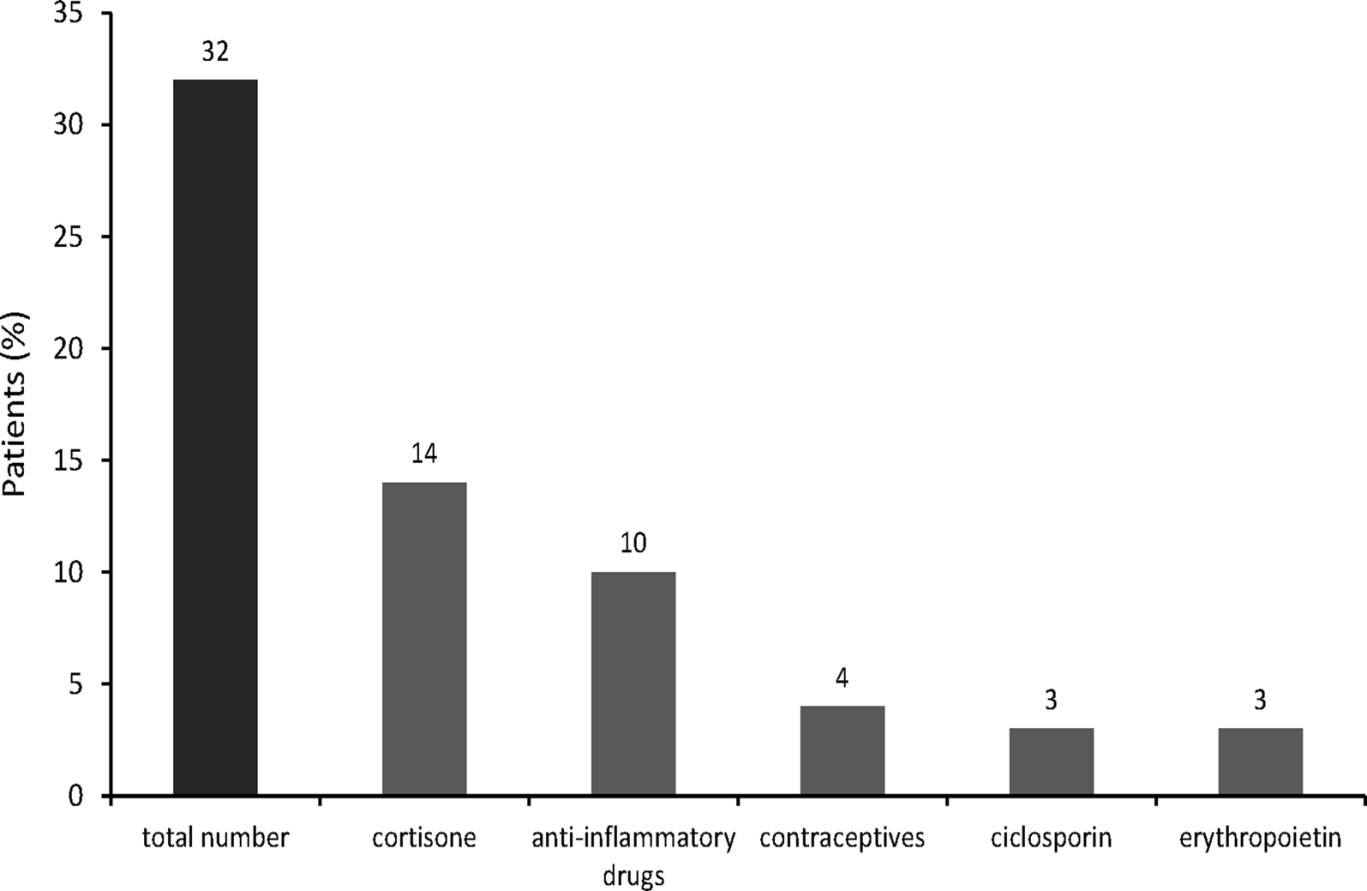
Percentage of patients receiving hypertension-inducing drugs

In addition to classical forms of secondary hypertension, we also collected data with regard to BP increasing medications. We found that 32 % of the subjects received drugs that could induce hypertension. These included mainly glucocorticoids, anti-inflammatory drugs (excluding aspirin, 100 mg), contraceptives, cyclosporins, and erythropoietins (Fig. 4).

### Cardio-renal co-morbidities

We observed that patients admitted and treated for arterial hypertension tended to be multi-morbid. Indeed, a large proportion of subjects displayed coronary heart disease (37 %) or renal insufficiency (56 %). Notably, 23 % of the patients had experienced a previous heart attack (13 %) or stroke (10 %). ECGs were performed in 85 patients at the time of admission (82.5 %). Of these, 79 (92.9 %) had a sinus rhythm, whereas 6 (7.1 %) had atrial fibrillation. The mean heart rate was 73.9 ± 15.7 bpm, with a maximum frequency of 118 bpm and a minimum frequency of 50 bpm. Echocardiography was performed on 55 patients (53.4 %), with 28 (50.9 %) displaying left ventricular hypertrophy and 8 (14.5 %) a decreased ejection fraction (<55 %).

### Medical treatment for hypertension

**Fig. 5:**
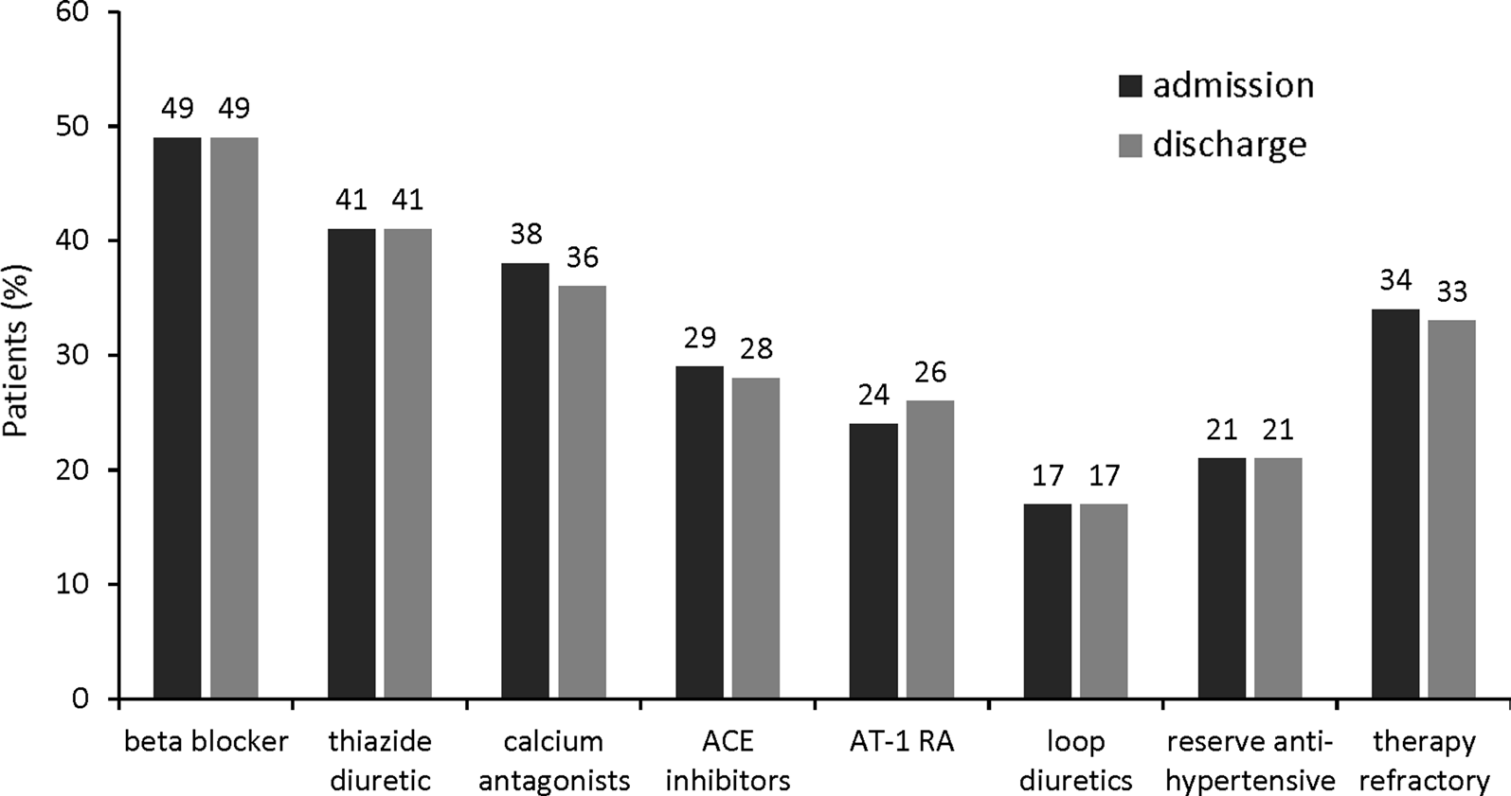
Distribution of patients based on the antihypertensive treatment on the day of admission and at discharge

We also collected data with regard to antihypertensive medications on the day of admission and at discharge (Fig. 5). Notably, a total of 34 % of our patients met the definition of therapy-refractory hypertension, receiving three or more antihypertensive drugs (including a diuretic). We found that the most frequently prescribed drugs in our inpatient population were beta blockers, followed by thiazide diuretics. Moreover, 21 % of the patients received reserve antihypertensive therapy, with the most commonly used drug being Moxonidin (12.6 %). This study did not examine patient adherence to the antihypertensive medication, but since 47.0 % of patients showed normotensive readings after admission (<140/90 mmHg), poor compliance can be assumed in many cases.

## Discussion

In the present study, we have examined the characteristics of individuals requiring inpatient treatment for arterial hypertension. Strikingly, we found that approximately half of our patients displayed normotensive readings upon hospital admission. Despite this finding, hypertension-related symptoms constituted the principal reason for hospitalization. Moreover, almost one-third of these patients could be classified as therapy refractory, whereas approximately two-thirds displayed secondary forms of hypertension.

### BP levels and the benefit of inpatient care

We observed lower than expected BP values on the day of admission. In fact, although the majority of our patients had presented with hypertension-related symptoms, only <10 % of them displayed grade 3 hypertension. However, since our BP measurements were documented after admission to the hospital ward, it is possible that emergency room BP values had been higher (i.e., reason for hospital admission). Indeed, lower BP following hospital admission could result from reduced stress levels or monitored intake of prescribed antihypertensive drugs (i.e., compliance) [15]. That being said, symptoms linked to hypertension have previously been reported to be unrelated to BP levels and instead linked with indirect factors, such as psychological issues (e.g., depression, anxiety), antihypertensive medications, and concomitant diseases [6–8]. Therefore, it is possible that this phenomenon may have also contributed to the lower than expected BP values in our admitted patients.

Inpatient treatment of our cohort led to improvements in BP. Indeed, we observed an approximately 50 % reduction in the number of patients with BP >140/90 mmHg at the time of discharge. This finding is interesting because it was previously shown that up to 77 % of inpatients treated for elevated BP remained hypertensive at the time of discharge and follow-up [14]. Nevertheless, our study was conducted within the Department of Nephrology and Rheumatology, which led to the inclusion of a high number of patients with renoparenchymatous hypertension. Thus, it is possible that the positive outcomes observed in the present study resulted from inpatient treatment of the large proportion of subjects displaying renal insufficiency and proteinuria (i.e., leading to reduced secondary hypertension in these individuals). Nevertheless, it is also plausible that other factors associated with inpatient care (e.g., improved adherence to medications) could also contribute to better short-term BP control in comparison to outpatient treatments [16, 17].

The expense associated with inpatient care is also an important consideration, as it has been suggested that outpatient care for hypertension is more cost effective [18]. Therefore, determining those patients that can benefit more from inpatient treatment could be highly advantageous from a cost perspective. In this regard, our findings represent an important step toward identifying such individuals. Taken together, our data preliminarily demonstrate the efficacy of inpatient treatment for hypertension, and suggest the need for additional studies to establish the specific patient groups that will benefit most from this therapeutic option.

### Secondary hypertension and hospitalization

It has been reported that approximately 5–10 % of adults with hypertension display an identifiable secondary cause [19]. However, in the present study we found that approximately two-thirds of our patient population presented with secondary hypertension. In this regard, the high proportion of patients with renoparenchymatous hypertension and/or treated with BP-boosting medications was striking. Therefore, it is possible that these characteristics are strongly associated with the need for inpatient treatment.

In this respect, the large proportion of individuals with renal insufficiency in our cohort might suggest that this comorbidity directly contributed to the need for hospital admission, and thus the probability of inpatient treatment for hypertension. Nevertheless, it is known that secondary forms of hypertension, especially kidney disease, are often associated with therapeutic resistance [20], which also could require inpatient intervention. In this respect, the relationship between comorbidities and hypertension-inducing drugs cannot be overlooked with regard to augmenting the severity of resistant forms of hypertension and the need for hospitalization. Thus, our results obtained from this highly comorbid population highlight the complex interplay that may exist between comorbidities, BP-boosting drugs, and therapy refractory hypertension. However, further study will be required to determine which specific characteristics are most strongly correlated with the requirement for inpatient treatment. In particular, raising awareness with regard to the role of hypertension-inducing drugs may be important for improving the management of these hypertensive patients. Indeed, our results suggest that more care may need to be taken when prescribing such drugs to high-risk patients.

Regardless of the reason for admission, our data preliminarily suggest that hospital supervision is especially beneficial for those with secondary forms of hypertension. Thus, inpatient care for these individuals may be necessary for efficient BP monitoring and control. Specific analysis of the different forms of secondary hypertension should yield information with regard to their unique associations with inpatient treatment as well as the benefit that can be achieved through this type of care.

### Resistant hypertension and anti-hypertensive medications

The exact prevalence of resistant hypertension is unknown, but it has been reported to occur in 5–50 % of cases [21]. In the present study, almost one-third of our patients was therapy refractory, suggesting that this characteristic may be linked to need for inpatient care. In addition, it was recently suggested that the prevalence of resistant hypertension is on the rise [22], which may mean that the establishment of specific guidelines for the effective management of hypertensive patients during hospitalization could be gaining importance. In this regard, the most effective combination therapies for effectively treating those with resistant hypertension remain to be determined [12]. Thus, identifying which antihypertensive drugs were most frequently administered to our hospitalized patients was interesting from a medical perspective. We found that beta blockers and thiazide diuretics were the most common drugs used in our patients, and that Moxonidin was the most often prescribed reserve antihypertensive (followed by Ebrantil). Although it is possible that the use of certain drugs could be linked with the need for inpatient treatment in hypertensive subjects, larger multi-center studies will be needed to provide evidence to link any specific treatments to the requirement for inpatient care. Indeed, beta blockers and thiazide diuretics are commonly employed for the treatment of hypertension, and Moxonidin has shown a good safety and efficacy profile [23]. The rate of beta-blocker use was high at 49 %. This may due to the high proportion of patients with cardiac diseases, with 37 % having coronary heart disease. Furthermore, the prevalence of therapy-resistant hypertension was 34 %, which corresponds to many patients being treated with more than three different anti-hypertensives, one of which is likely to have been a beta-blocker.

### Study limitations

This study presented several limitations. In particular, our cohort of patients may not have been large enough to draw significant conclusions regarding patient characteristics associated with inpatient treatment of hypertension. Nevertheless, the present study represents a first step in determining which patients require inpatient care as well as the value of this treatment option. In addition, the fact that our investigation was conducted specifically within the Department of Nephrology and Nephrology at a single institution may have created bias. Indeed, there was a large number of patients with renal insufficiency included in this study. Thus, future multi-center investigations involving different departments will be required to thoroughly assess the diverse patient characteristics that may be related to the need for inpatient therapy for hypertension. Moreover, the lack of direct comparison between our cohort and outpatients is a limitation of our study. Indeed, in order to truly determine the characteristics that define patients treated on an inpatient basis, analysis of outpatients from the same institution will be required. In this regard, future studies aimed at assessing the benefit of inpatient care as compared to outpatient treatment options will be required to fully assess specific characteristics and therapeutic responses in hypertensive patients.

## Conclusions

For the most part, patients with arterial hypertension were hospitalized due to their clinical symptoms and not as a result of BP values alone. The high proportion of patients with secondary forms of hypertension or treated with BP-boosting medications was striking. In addition, a large percentage of patients displayed resistant hypertension. Thus, it is possible that these factors were associated with the need for inpatient treatment as a result of high BP. Taken together, these findings can contribute to improve the clinical management of hypertensive patients. However, additional investigations will be needed to more thoroughly examine the relationship between these characteristics and inpatient care as well as to verify that reductions in BP can be achieved through brief inpatient treatment.

## Abbreviations

BP: blood pressure
BMI: body mass index
CKD: chronic kidney disease
CHD: coronary heart disease

## References

[1] World Heart Federation. Cardiovascular disease risk factors: hypertension. Internet: http://www.world-heart-federation.org/cardiovascular-health/cardiovascular-disease-risk-factors/hypertension/. 2014. Accessed on 10. Mar 2014.

[2] World Health Organization (WHO). A global brief on hypertension: silent killer, global public health crisis. WHO reference number: WHO/DCO/WHD/2013.2. April 2013.

[3] Egan BM, Zhao Y, Axon RN (2010). US trends in prevalence, awareness, treatment, and control of hypertension, 1988–2008. JAMA.

[4] Sarafidis PA, Li S, Chen SC, Collins AJ, Brown WW, Klag MJ, Bakris GL (2008). Hypertension awareness, treatment, and control in chronic kidney disease. Am J Med.

[5] Salkic S, Batic-Mujanovic O, Ljuca F, Brkic S (2014). Clinical presentation of hypertensive crises in emergency medical services. Mater Sociomed.

[6] Bulpitt CJ, Dollery CT, Hoffbrand BI (1977). The contribution of psychological features to the symptoms of treated hypertensive patients. Psychol Med.

[7] Karras DJ, Ufberg JW, Harrigan RA, Wald DA, Botros, McNamara RM (2005). Lack of relationship between hypertension-associated symptoms and blood pressure in hypertensive ED patients. Am J Emerg Med.

[8] Cooper WD, Glover DR, Hormbrey JM (1988). Symptoms in hypertensive patients: the effect of treatment withdrawal. J Hypertens Suppl.

[9] Grossman E, Messerli FH (2012). Drug-induced hypertension: an unappreciated cause of secondary hypertension. Am J Med.

[10] Streeten DH, Anderson GH (1992). Secondary hypertension. An overview of its causes and management. Drugs.

[11] Acelajado MC, Pisoni R, Dudenbostel T, Dell’Italia LJ, Cartmill F, Zhang B (2012). Refractory hypertension: definition, prevalence, and patient characteristics. J Clin Hypertens (Greenwich).

[12] Kumar N, Calhoun DA, Dudenbostel T (2013). Management of patients with resistant hypertension: current treatment options. Integr Blood Press Control.

[13] Calhoun DA, Jones D, Textor S, Goff DC, Murphy TP, Toto RD (2008). Resistant hypertension: diagnosis, evaluation, and treatment. A scientific statement from the American Heart Association Professional Education Committee of the Council for High Blood Pressure Research. Hypertension.

[14] Axon RN, Cousineau L, Egan BM (2011). Prevalence and management of hypertension in the inpatient setting: a systematic review. J Hosp Med.

[15] Jung O, Gechter JL, Wunder C, Paulke A, Bartel C, Geiger H, Toennes SW (2013). Resistant hypertension? Assessment of adherence by toxicological urine analysis. J Hypertens.

[16] Mazzaglia G, Ambrosioni E, Alacqua M, Filippi A, Sessa E, Immordino V (2009). Adherence to antihypertensive medications and cardiovascular morbidity among newly diagnosed hypertensive patients. Circulation.

[17] Vrijens B, Vincze G, Kristanto P, Urquhart J, Burnier M (2008). Adherence to prescribed antihypertensive drug treatments: longitudinal study of electronically compiled dosing histories. BMJ.

[18] Wagner AK, Valera M, Graves AJ, Laviña S, Ross-Degnan D (2008). Costs of hospital care for hypertension in an insured population without an outpatient medicines benefit: an observational study in the Philippines. BMC Health Serv Res.

[19] Viera AJ, Neutze DM (2010). Diagnosis of secondary hypertension: an age-based approach. Am Fam Physician.

[20] Faselis C, Doumas M, Papademetriou V (2011). Common secondary causes of resistant hypertension and rational for treatment. Int J Hypertens.

[21] Sarwar MS, Islam MS, Al Baker SM, Hasnat A (2013). Resistant hypertension: underlying causes and treatment. Drug Res (Stuttg).

[22] Roberie DR, Elliott WJ (2012). What is the prevalence of resistant hypertension in the United States?. Curr Opin Cardiol.

[23] Schachter M (1999). Moxonidine: a review of safety and tolerability after seven years of clinical experience. J Hypertens Suppl.

